# Resolving the problem of trapped water in binding cavities: prediction of host–guest binding free energies in the SAMPL5 challenge by funnel metadynamics

**DOI:** 10.1007/s10822-016-9948-6

**Published:** 2016-08-29

**Authors:** Soumendranath Bhakat, Pär Söderhjelm

**Affiliations:** Division of Biophysical Chemistry, Chemical Center, Lund University, P.O.B. 124, 22100 Lund, Sweden

**Keywords:** SAMPL5, Host–guest complex, Metadynamics, Binding free energy, MD simulation

## Abstract

The funnel metadynamics method enables rigorous calculation of the potential of mean force along an arbitrary binding path and thereby evaluation of the absolute binding free energy. A problem of such physical paths is that the mechanism characterizing the binding process is not always obvious. In particular, it might involve reorganization of the solvent in the binding site, which is not easily captured with a few geometrically defined collective variables that can be used for biasing. In this paper, we propose and test a simple method to resolve this trapped-water problem by dividing the process into an artificial host-desolvation step and an actual binding step. We show that, under certain circumstances, the contribution from the desolvation step can be calculated without introducing further statistical errors. We apply the method to the problem of predicting host–guest binding free energies in the SAMPL5 blind challenge, using two octa-acid hosts and six guest molecules. For one of the hosts, well-converged results are obtained and the prediction of relative binding free energies is the best among all the SAMPL5 submissions. For the other host, which has a narrower binding pocket, the statistical uncertainties are slightly higher; longer simulations would therefore be needed to obtain conclusive results.

**Electronic supplementary material:**

The online version of this article (doi:10.1007/s10822-016-9948-6) contains supplementary material, which is available to authorized users.

## Introduction

Prediction of the binding free energy of a protein–ligand complex using computational methods is one of the most important research topics in structure-based drug development. It is a challenging problem; even the most advanced methods cannot quantitatively predict the binding free energy for a range of systems [1]. All methods based on molecular dynamics (MD) simulations share the problem of limited accuracy of the underlying force field. In addition, they all suffer from insufficient conformational sampling due to the timescale issue. However, the methods typically differ in their ability to sample different types of degrees of freedom relevant for ligand binding.

Several types of statistically rigorous free-energy methods have been developed [2]. *Alchemical perturbation* methods, such as free-energy perturbation, evaluate the binding free energy via an artificial unphysical path involving creation and deletion of atoms. They typically give well converged free energy estimates, especially for relative free energies of similar ligands. In practice, they often require that a well-defined binding pose is known, because the time required for sampling multiple poses in unbiased MD simulations is typically very long. In contrast, *potential of mean force* (PMF) methods simulate the movement (and derives the PMF profile) along a physically realizable (but not necessarily optimal) binding path. The advantage of such methods is that they might overcome sampling problems more easily and that they produce a more complete picture of the energy landscape of the complex; if the optimal binding path is found, they even give some estimate of the height of kinetic barriers along the path. Due to their computational cost, application of rigorous free-energy methods to large-scale data sets has been rather limited. In contrast, the more approximate MM/PBSA and MM/GBSA approaches are often used at a larger scale, but their reliance on implicit-solvent models and their inadequate treatment of entropy make their performance highly system dependent [3].

Several methods have been devised to enhance sampling along specific coordinates. Metadynamics [4, 5] is one of the enhanced-sampling approaches gaining popularity for predicting binding poses and binding free energies [6]. Together with e.g. umbrella sampling, it belongs to the class of PMF methods. It is especially well suited for systems in which there are multiple binding poses [7, 8] or in which the binding pose is not known a priori [9]. In order to be a rigorous method, metadynamics requires a set of predefined collective variables (CVs) representing all the slow degrees of freedom of the system, but the method has also been used in more approximate ways to improve docking poses [10, 11]. In accurate metadynamics studies of protein–ligand binding, the collective variables typically include the protein–ligand distance, some descriptor of the orientation of the ligand relative to the protein, and sometimes an additional variable describing a conformational change in the protein [8]. To avoid that the ligand spends too much time exploring the unbound state, it has to be restrained to a certain region of the protein surface. A particularly efficient variant of this approach is the *funnel metadynamics* method, which applies a funnel-shaped restraint potential that reduces the explored space outside the binding site to a narrow tube while not affecting the exploration of the binding site significantly [12]. Recently, the funnel metadynamics method has been applied to a variety of problems, including host–guest systems [13], the binding mechanism of a neurotransmitter to a ligand-gated ion channel [14], and the reproduction of NMR observables in the binding of small ligands to peroxiredoxin [15, 16].

The dynamics of water molecules in the binding cavity is a key factor in the ligand-binding kinetics [17]. In practical terms, the handling of these water molecules is a general consideration that applies to all types of ligand-binding methods, but it manifests itself in different ways for the various methods, e.g. for MM/PBSA [18], alchemical perturbation [19], and PMF methods [20]. In metadynamics, which depends on a multitude of binding and unbinding events for obtaining an accurate PMF, water molecules that become trapped in the binding site and hinder the entry of the ligand can severly affect the convergence rate of the method by causing a hysteresis effect. The problem has sometimes been discussed, and special collective variables have been devised [9], but more often the problem is simply considered as one of many factors slowing down the convergence of metadynamics. The extent of the problem depends on the geometry of the binding cavity; if the ligand channel is wide or there is a “second exit” that the water molecules can use to avoid the clash with the entering ligand, the problem is negligible.

Historically, free-energy methods have been validated against experimental binding free energies using existing data sets. Such tests are vulnerable to experimental or analytical bias, and even though most studies carefully avoid these issues, it is unavoidable that, in the published validation literature as a whole, there is a bias towards good agreement with experiment, simply based on the tendency of scientific journals to preferentially publish success stories. In that respect, blind predictions such as the *Statistical Assessment of the Modeling of Proteins and Ligands* (SAMPL) play a pivotal role for evaluating the true predictive power of free-energy methods, conformational sampling procedures, and force fields. Indeed, the results for protein–ligand binding in these challenges have been somewhat disappointing, with the best results often obtained with empirical scoring functions [21]. In two previous SAMPL challenges, binding free energy prediction of smaller *host–guest* complexes has been used to test methods designed for protein–ligand binding [22, 23]. The host molecules, being much smaller than proteins, allow testing of expensive computational methods without wasting too much computer time. Moreover, these systems do not have problems like metal parameterization, conformational flexibility, residue flipping, and uncertain protonation states, while still displaying mainly the same types of interactions. This makes them perfect candidates for a thorough testing of methods, although subsequent tests on real biological systems, with their additional challenges, are also necessary.

In this study, we use funnel metadynamics to study the binding of a set of six guest molecules to two octa-acid hosts as part of the SAMPL5 challenge [24]. The funnel metadynamics method has previously been used for predicting host–guest binding free energies in the SAMPL4 challenge [13]. In that study, good statistical precision was obtained, but due to a non-optimal choice of force field, no agreement with experiment was obtained. Moreover, long simulation times (100–200 ns per ligand) were needed for convergence. Therefore, we here apply the same method in the SAMPL5 challenge, although with more commonly used force fields, and while simultaneousy trying to reduce the required simulation time. The shape of the octa-acid host molecules is also quite different from the cucurbit[7]uril host studied previously; in particular one can expect effects of trapped water in the octa-acid host because of its deep and narrow binding cavity.

The aim of the study is threefold: First, we establish an accurate and efficient protocol for calculating binding free energies with funnel metadynamics, including the handling of the trapped-water problem. Second, we test the whole methodology, including the force field and system setup, in a “blind” manner through the SAMPL5 procedure. Finally, because host–guest systems are in many senses considered “easy” systems to model (because of the small number of accessible conformations), we also test how well the much simpler MM/PBSA approach performs for the same systems.

## Methods

### System preparation and simulation details

**Fig. 1:**
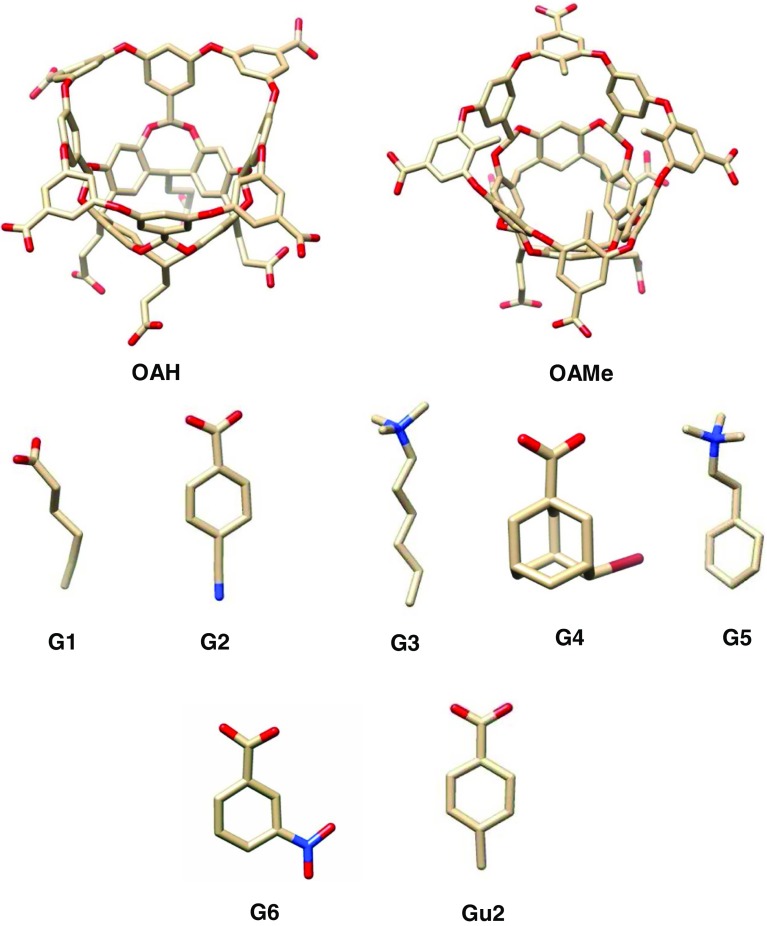
Graphical representation of the two octa-acid hosts, OAH (without methyl groups) and OAMe (with methyl groups), along with the guest molecules *G1*–*G6* and *Gu2*, which are used in this study. The *OAH*–*Gu2* complex is not part of the SAMPL5 data set but is only used as a reference to establish an approximate level for the absolute binding free energies

We study the binding of 6 small molecules to two different octa-acid host systems, as shown in Fig. 1. The host OAH was previously used in the SAMPL4 challenge, whereas the host OAMe is identical to OAH except for the addition of four methyl groups which alter its cavity shape and hydrophobicity. To investigate the dependence of the results on the force field, two different force fields were employed in the calculations: the General Amber force field (GAFF) [25] and the OPLS force field [26].

#### GAFF

Simulations were performed with Gromacs 4.6.2 [27] and the GAFF force field [25] with RESP charges [28] for the hosts and guests, and the TIP3P water model [29], using the simulation files supplied in the SAMPL5 package. The system thus contained the host, the guest, 7–9 sodium ions (in order to neutralize the systems) and $\sim$2100 water molecules. The temperature was kept at 298 K using the velocity-rescaling algorithm [30]. All bonds lengths were constrained using the LINCS algorithm to enable a timestep of 2 fs. A nonbonded cutoff of 1.5 nm was used, and long-range electrostatics were treated by Particle mesh Ewald summation (PME) using a grid spacing of 0.1 nm. Each system (bound conformation) was first equilibrated in the NPT ensemble (i.e. using constant pressure and temperature) using the Berendsen barostat for at least 5 ns, and a snapshot with a volume near the average of this ensemble was used as a starting point for the metadynamics simulations ($\sim$70 ns for each of the 13 systems), which were performed in the NVT ensemble (i.e. using constant volume and temperature).

Unbiased MD simulations of the complexes (of length 40 ns) for use with MM/PBSA were also performed in the NVT ensemble after 5 ns of NPT equilibration. Unbiased MD simulations of the free hosts for analyzing the solvation behavior (length 20 ns) were started from the complex structure after removal of the guest molecule and adjusting the number of sodium ions; these were also performed in the NVT ensemble after 5 ns of NPT equilibration.

#### OPLS

Another set of simulations were performed with Gromacs 4.6.2 and the OPLS-AA force field [26] for the hosts and guests and the TIP3P water model. The parameters (including partial charges) for the host and guest molecules were obtained through the ffld_server module of the academic package of Maestro [31], and converted to Gromacs using the ffconv.py script [32]. All simulation settings and equilibration procedures were identical to the simulations with the GAFF force field.

### Funnel metadynamics

**Fig. 2:**
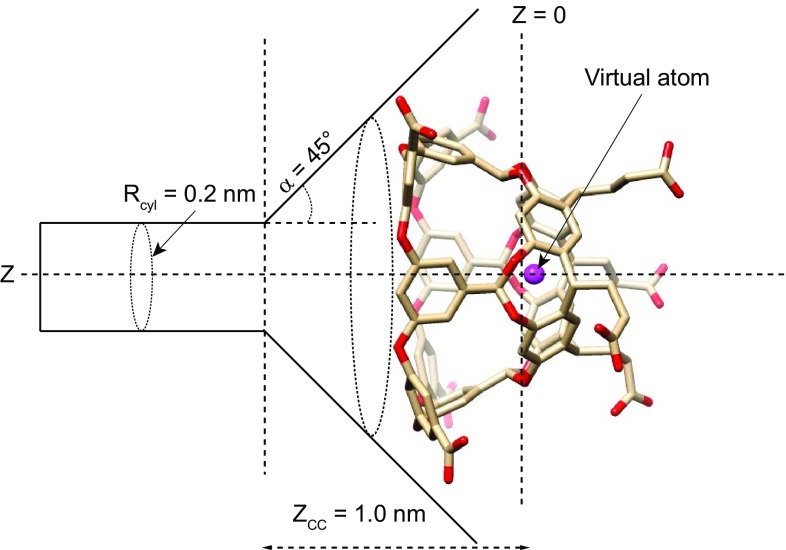
Geometry of the system setup showing the definition of the host axis (*z*) and the shape and location of the funnel potential (Eq. 1) used in all funnel metadynamics simulations. The virtual atom used for defining the water restraint in Eq. 3 is also shown

Funnel metadynamics is an enhanced sampling approach which enhances the sampling along the ligand binding path and applies a funnel-shaped restraint potential that reduces the space to explore in the unbound state. The PLUMED 1.3 plugin for free-energy calculations [33] was used for introducing all biasing potentials in this study. Two collective variables were defined: the first collective variable (CV 1) is defined as the projection *z* of the ligand’s centre-of-mass upon the host’s C_4_ symmetry axis, as shown in Fig. 2. The second collective variable (CV 2) equals, for a rigid ligand, the cosine of the angle $\theta$ between the host axis and a guest molecular axis defined individually for each guest, as depicted in Fig. S1 in the supporting information. For a flexible ligand, CV 2 can be somewhat influenced by the conformation; the exact definition follows an earlier publication [13]. The purpose of CV 1 is to accelerate the movement of the guest into and out of the host, whereas the purpose of CV 2 is to promote exploration of the rotational space of the ligand and, to some extent, also the conformational space in case of a non-rigid ligand.

The funnel potential is depicted in Fig. 2. Using the notation of Ref. [12], the following parameters was used: $z_{cc} = 1.0$ nm, $R_{cyl} = 0.2$ nm, and $\alpha =$ 45$^\circ$. More precisely, if $R_f(z)$ is the radius of the funnel at a given *z*, the restraint potential for the guest at a centre-of-mass distance *r* from the *z* axis equals

1 $$\begin{aligned} V_f(r,z)= {\left\{ \begin{array}{ll} \kappa \left[ r-R_f(z)\right] ^2, &{} \quad \text {if }\, r>R_f(z) \\ 0, &{} \quad \text {otherwise,} \end{array}\right. } \end{aligned}$$

where $\kappa =478$ kcal mol$^{-1}$ nm$^{-2}$. Finally, a steep repulsive wall was applied on *z*, acting above $z_ {\text {wall}} \approx 1.85$ nm (the exact value varied slightly with the size of the guest molecule) with the functional form $V_ {\text {wall}} =k_ {\text {wall}} (z-z_ {\text {wall}} )^4$, where $k_ {\text {wall}} =1.195 \times 10^{5}$ kcal mol$^{-1}$ nm$^{-4}$, in order to prevent the guest molecule from interacting with other periodic images of the host.

The time-dependent metadynamics bias potential was deposited in the two-dimensional space described by CV 1 and CV 2, with gaussian widths of 0.04 nm and 0.043, respectively, and a hill-deposition stride of 1.0 ps. The well-tempered formulation of metadynamics was used [34], with an initial hill height of 0.1195 kcal/mol and a bias factor of 20.

At a given time of simulation, the binding free energy was calculated as follows. First, the two-dimensional free energy surface was projected down on CV 1 (*z* axis) using a Boltzmann averaging over CV 2 at a temperature of 298 K. The reconstruction of the free energy surface and the projection were both performed using the sum_hills utility provided with the PLUMED plugin [33].

As discussed in Ref. [2], the free energy difference between the bound and unbound state ($\varDelta G_{meta}$) is related to the one-dimensional PMF *w*(*z*) through

2 $$\begin{aligned} \mathrm {e}^{-\beta \varDelta G_{meta}}=C^0 S^*\mathrm {e}^{\beta \varDelta G_a^{site}} \int _{\mathrm {site}}dz\mathrm {e}^{-\beta \left[ w(z)-w(z^*)\right] }\text { ,} \end{aligned}$$

where $C^0 = 1/1.66$ nm$^{-3}$ is the standard concentration, $S^*$ is the effective cross-sectional area swept by the ligand restrained along the *z* axis, $\varDelta G_a^{site}$ is the free energy for restraining the bound ligand along the axis, and $\beta =(k_B T)^{-1}$ where $k_B$ is Boltzmann’s constant and *T* is the absolute temperature. In our case, the restraint does not affect the bound ligand due to its funnel shape, so $\varDelta G_a^{site}=0$. Moreover, owing to the steep shape of $V_f$, we assume that the ligand motion in the unbound state is restricted to the flat region, so that $S^*=\pi R_{cyl}^2$. These are the same assumptions as done in Refs. [12, 13]. On evaluating Eq. 2, we define the *site* as $z<0.9$ nm (we verified that changing this limit between 0.8 and 1.0 nm affected the result by less than 0.1 kcal/mol for all molecules). The reference level of the PMF, $w(z^*)$, corresponding to the unbound state, was evaluated as the average *w*(*z*) over the range $1.4\le z \le 1.8$ nm.

Preliminary metadynamics simulations showed significant hysteresis attributed to the slow displacement of water molecules when the guest molecule reentered the host. To avoid this problem, we model the unbound conformation in a “dry state”, for which the water molecules are artificially prevented from entering the inner part of the host molecule by means of a restraint. The restraint takes the form of a steep wall acting on a generalized water coordination number *S* of a virtual atom located at the centre of mass of the deepest-lying phenyl rings (see Fig. 2):

3 $$\begin{aligned} V(S)= & {} {\left\{ \begin{array}{ll} k_s[S-S_0]^4,&{} \quad \text {if } \, S\ge S_0\\ 0, &{} \quad \text {otherwise, and} \end{array}\right. } \end{aligned}$$

4 $$\begin{aligned} S= & {} \sum _i \frac{1-\left( \frac{r_{iv}}{r_0}\right) ^n}{1-\left( \frac{r_{iv}}{r_0}\right) ^m}\text { ,} \end{aligned}$$

where $k_s=2.39 \times 10^5$ kcal/mol, $S_0=0.02$, $n=16$, $m=32$, $r_0=0.35$ nm, and the sum goes over all water molecules in the system, with $r_{iv}$ being the distance between the oxygen atom of molecule *i* and the virtual atom inside the host. The steep switching function in Eq. 4 ensures that the restraint has no effect on other water molecules than those immediately trying to enter the cavity.

**Fig. 3:**
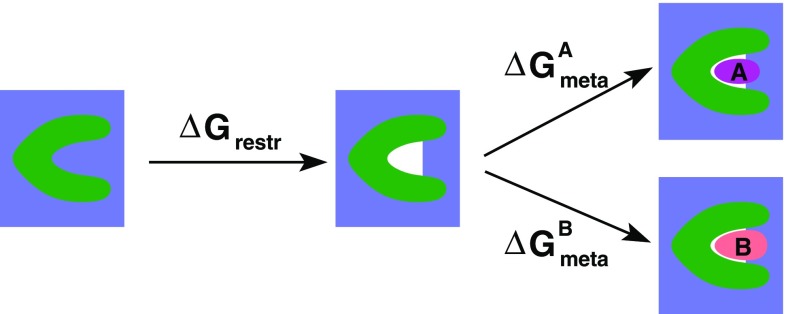
Schematic picture of the subdivision of the binding free energy into an artificial desolvation part ($\varDelta G_ {\text {restr}}$), which is independent of the ligand, and the part computed by funnel metadynamics ($\varDelta G_ {\text {meta}}$)

The procedure is schematically depicted in Fig. 3. The introduction of the dry state as end state for the metadynamics simulations is expected to have a significant effect ($\varDelta G_ {\text {restr}}$ ) on the absolute binding free energy, but it is identical for all ligands, because the restraint is designed not to affect the bound state. For a standard MD simulation of the bound state, $V(S)=0$ about 98 % of the time, as demonstrated in Fig. S3 in the supporting information.

The cost $\varDelta G_ {\text {restr}}$ of introducing the water restraint can be rigorously evaluated by free-energy perturbation (exponential averaging) [35]. In general, this transformation may need to be subdivided into many smaller steps, but as will be shown in the results section, such subdivision was not necessary for the current system, because the dry state was frequently visited during the unrestrained MD simulation of the host. Thus, for each host, the restraint contribution was calculated as

5 $$\begin{aligned} \varDelta G_ {\text {restr}} =-k_BT \ln \langle \exp \left( -V(S)/k_BT \right) \rangle _{0} \text { ,} \end{aligned}$$

where the subscript 0 denotes that the average is taken over the unrestrained ensemble. The *V*(*S*) data taken from a 20 ns MD simulation was first filtered by computing the statistical inefficiency of each time series and taking data points separated by this amount of time (6–43 ps) to ensure statistically independent samples. To estimate the resulting statistical precision, a bootstrapping procedure with 100 repeats was used. The standard deviation of the obtained set of $\varDelta G_ {\text {restr}}$ values is reported as the statistical error.

The total binding free energy is computed as

6 $$\begin{aligned} \varDelta G_ {\text {bind}} = \varDelta G_ {\text {meta}} + \varDelta G_ {\text {restr}} \end{aligned}$$

and the statistical uncertainty is dominated by the $\varDelta G_ {\text {meta}}$ contribution. The $\varDelta G_ {\text {meta}}$ obtained from Eq. 2 was monitored regularly for convergence. A time average over the last 20 ns of the simulation was used to obtain the final results [5] and the statistical uncertainty was calculated as the maximum of two numbers: the fluctuation of the $\varDelta G_{meta}$ estimate during this period and the absolute difference between the averages of the two last consecutive blocks of 20 ns (to account for possible drift).

With the GAFF force field, two sets of results are presented, our original SAMPL5 submission and the refined result. The two sets differ in two respects. In the submitted result, the free energy was averaged over the last 10 ns of a $\sim$40 ns run (in contrast to the last 20 ns of a longer $\sim$70 ns run for the refined result). Second, for our SAMPL5 submission, we only extracted relative energies from the metadynamics calculations, because we did not have converged values for the $\varDelta G_ {\text {restr}}$ contribution at the deadline of submission. In order to submit absolute estimates, we included a complex from the SAMPL4 challenge with known binding free energy in the set of calculations, and adjusted the estimated binding free energies with an empirical constant $\varDelta G_ {\text {emp}} =5.86$ kcal/mol, i.e.

7 $$\begin{aligned} \varDelta G_ {\text {bind}} ^ {\text {submitted}} = \varDelta G_ {\text {meta}} + \varDelta G_ {\text {emp}} \text { ,} \end{aligned}$$

so that this known experimental value, $-5.9$ kcal/mol, was reproduced [23, 36]. This complex consisted of the OAH host and ligand 2 from SAMPL4, and thus will be called OAH–Gu2 in the following.

### MM/PBSA calculations

The binding free energy for both GAFF and OPLS parametrized systems was also calculated using the MM/PBSA approach as implemented in the standalone version of the g_mmpbsa tool [37]. The binding free energy was calculated from an unrestrained MD simulation of $\sim$40 ns for each host–guest complex. For protein–ligand systems, it has been suggested that many shorter simulations should be run instead [38], but this was not considered important for the current systems as they displayed very little conformational variation. Snapshots from the simulation were collected every 10 ps, and stripped from water and counter ions. The MM-PBSA energy was computed for each snapshot as a sum of three terms:

8 $$\begin{aligned} \varDelta G_ {\text {bind}} ^ {\text {MM/PBSA}} = E_ {\text {MM}} ^ {\text {int}} + \varDelta G_ {\text {PB}} + \varDelta G_ {\text {np}} \text { .} \end{aligned}$$

Here, $E_ {\text {MM}} ^ {\text {int}}$ is the MM interaction energy (sum of electrostatics and van der Waals energy) between the host and the guest. $\varDelta G_ {\text {PB}}$ is the change in Poisson-Boltzmann solvation energy upon binding, evaluated using zero ionic strength, a solute dielectric constant of 1, a water dielectric of 80, a solvent probe radius of 1.4 Å, the *smol* model for construction of the cavity, Bondii radii, and otherwise default parameters of the g_mmpbsa software. Finally, $\varDelta G_ {\text {np}}$ is the nonpolar solvation energy, based on the change in solvent-accesible surface area (SASA) upon binding:

9 $$\begin{aligned} \varDelta G_ {\text {np}} =\gamma \varDelta (SASA) + \delta \text { ,} \end{aligned}$$

where $\gamma =5.43$ kcal mol$^{-1}$ nm$^{-2}$, $\delta =0.92$ kcal/mol, and the SASA was estimated using a probe radius of 0.14 nm and default parameters of the g_mmpbsa software. The reported values are the averages over all snapshots. The statistical error is estimated as the standard error of the mean, after having validated that the samples are statistically independent. Note that only relative binding free energies can be evaluated due to the absence of the entropy term from the calculations.

## Results and discussion

We performed well-tempered funnel metadynamics (WT–FM) and MM/PBSA analyses for each of the six guests G1–G6 against the two octa acid hosts, OAH and OAMe (and for the reference compound Gu2 binding to OAH). The funnel metadynamics simulations were performed using two collective variables: the projection of the ligand’s centre of mass upon the host’s C$_4$ symmetry axis, and the cosine of the angle $\theta$ between the host axis and the guest axis.

### Convergence

**Fig. 4:**
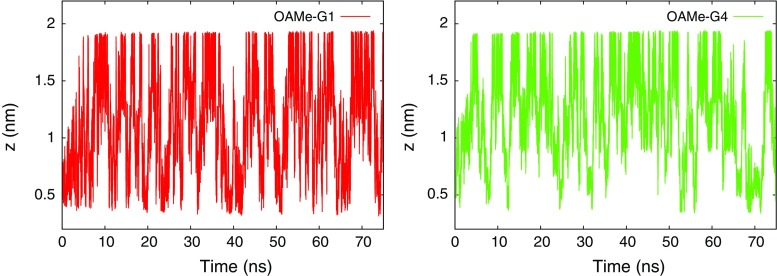
Time evolution of CV 1 (projecion of the ligand on the *z* axis) during the WT–FM simulations of the OAMe–G1 complex (*left*) and the OAMe–G4 complex (*right*) using the GAFF force field. The bound state is characterized by $z=0.5$–0.6 nm, whereas the unbound state has $z>1.3$ nm. The results for the other systems resemble the left figure; the OAMe–G4 complex is exceptional in that it shows fewer visits to the bound state

**Fig. 5:**
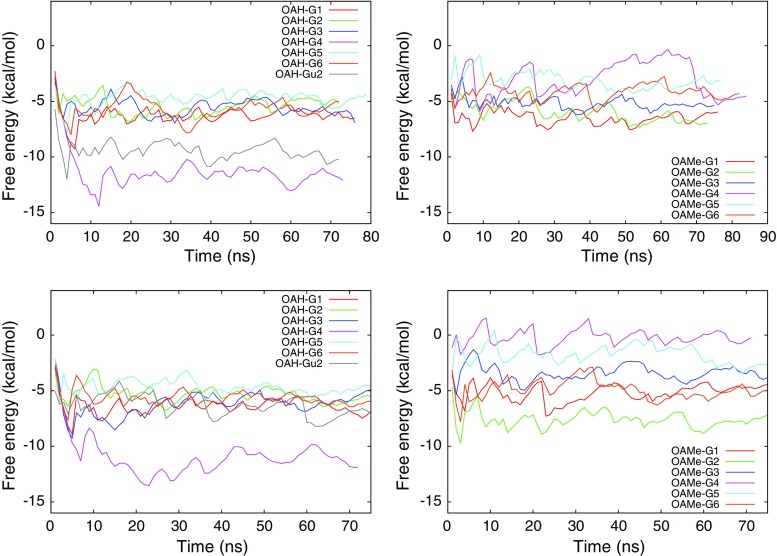
Time evolution of the free energy estimate ($\varDelta G_ {\text {meta}}$; in kcal/mol) during the WT–FM simulations for both OAH and OAMe host–guest systems using the GAFF force field (*upper row*) and the OPLS force field (lower row). After a transient time (less than 30 ns), each curve, except for the problematic OAMe–G4 complex, shows an oscillating behavior with a rather short period (less than 20 ns)

During the WT–FM of all systems, several recrossings occurred between the bound and unbound state, as demonstrated in Fig. 4. The presence of the funnel potential promotes the transition between the bound and unbound states by limiting the explored space in the unbound state. The convergence of the free energy surface plays an important role in deciding the statistical precision of the results obtained from WT–FM. Figure 5 shows the time evolution of the estimate of $\varDelta G_ {\text {meta}}$ (Eq. 2) for GAFF and OPLS parameterized systems. To reduce the statistical noise, a time average over the last 20 ns was used; this interval was determined to be sufficiently long to include several binding and unbinding events, while being sufficiently short to exclude the transient build-up of the bias potential in the beginning of the simulation. Table S1 in the supporting information provides a validation of this procedure by reporting block averages of $\varDelta G_ {\text {meta}}$ over several time periods. The fluctuation of the $\varDelta G_ {\text {meta}}$ estimate is 0.5 kcal/mol on average, and the average absolute difference between taking the results in the 30–50 ns interval or in the 50–70 ns interval is also 0.5 kcal/mol, but because there is significant variation among the systems, we define an error estimate for each system as the maximum of these two numbers. This estimate lies in the range 0.3–0.9 kcal/mol for all but two systems: OAMe–G4 with GAFF and OAMe–G5 with OPLS. In particular, the OAMe–G4 complex shows the worst convergence behavior. The bulky G4 molecule experimentally proved to be a weak binder to OAMe and it has difficulties in finding its way back into the deep binding cavity of OAMe during WT–FM. As shown in Fig. S5 in the supporting information, the convergence can be slightly improved by choosing another molecular axis for defining CV 2. We conclude that, with the exception of OAMe–G4 and possibly OAMe–G5, all results are statistically converged within $\sim$1 kcal/mol.

### Contribution from the water restraint

As detailed in the method section, we employ a restraint that prevents water molecules from entering the deeper part of the host while the ligand is in the unbound state in the metadynamics simulations. As shown in Fig. S2 in the supporting information, the bound state is much more frequently visited with this restraint present (by a factor 5–10), thus leading to a significantly faster convergence. In fact, without the restraints, the visits to the bound state become even less frequent with time, a typical behavior of well-tempered metadynamics when there is a kinetic barrier that is not addressed with the collective variables. It is notable that, without restraints, the host almost always contains inner water molecules ($S\ge 1$), except for the periods when the ligand is bound. This indicates that these water molecules constitute the barrier, and the improvement when adding the water restraint provides further evidence in this direction.

As shown in Fig. S4 in the supporting information, the “dry” state occurred frequently in normal unrestrained MD simulations of the host (2–10 % of the time, with the lowest numbers for OAH). This fact allowed evaluation of $\varDelta G_ {\text {restr}}$ by the one-step FEP method (Eq. 5). The results are shown in Table 1. The $\varDelta G_ {\text {restr}}$ contribution is $\sim 3$ kcal/mol for OAH and $\sim 2$ kcal/mol for OAMe, with uncertainties up to 0.2 kcal/mol. This contribution is included in the $\varDelta G_ {\text {bind}}$ energies discussed below.

**Table 1:** Free-energy contribution in kcal/mol from the water restraint ($\varDelta G_ {\text {restr}}$), calculated through FEP using 20 ns of MD simulation for each host and force field (FF), and the corresponding statistical error estimated through a bootstrapping procedure

Host	FF	$\varDelta G_ {\text {restr}}$	$\tau$ (ps)
OAH	GAFF	2.66 ± 0.10	7
OAH	OPLS	2.63 ± 0.24	30
OAMe	GAFF	1.76 ± 0.14	43
OAMe	OPLS	2.02 ± 0.13	40

The statistical inefficiency $\tau$ of the *V*(*S*) time series was only used to filter the data

### Free-energy profiles

**Fig. 6:**
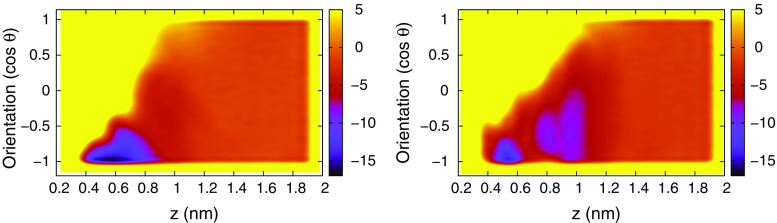
Two dimensional free energy surface (with the arbitrary zero level aligned with the unbound state and with the *color* scale in kcal/mol, with *yellow* representing larger values than 5 kcal/mol, including unsampled regions) for the GAFF-parameterized OAMe–G2 complex (*left*) and OAMe–G4 complex (*right*). For consistency with the reported results, the FES is averaged over the last 20 ns of the simulation. The results for the remaining complexes are qualitatively similar to the left picture, and the results for the two force fields are similar. The funnel restraint, acting outside the host on another coordinate, does not influence the qualitative features of these free-energy surfaces (any resemblance of a funnel is incidental)

Two examples of two-dimensional free-energy surfaces (FES) obtained from the WT–FM calculations are shown in Fig. 6. The FES is rather featureless, with a flat region with *z* between 1.4 and 1.8 nm, independent of $\theta$, and a binding site at $z=0.4$–0.6 nm and $\cos \theta \approx -1$, i.e. with the guest axis rather well aligned with the host axis, with the charged group pointing out into the solvent. The opposite orientation ($\cos \theta > 0$) of the bound guest has much higher free energy and is not sampled at our choice of bias factor; however, in the contact region around $z\approx 1.0$ nm, the full range of orientations are sampled, and preliminary calculations showed that the enhanced sampling of the guest orientation in this region is crucial for facilitating entry into the host and thereby obtaining convergence of the FES. For the G4 guest (and no others), a secondary minimum is seen in the contact region, but because the minima are well separated on the *z* axis, there is no fundamental loss of information when integrating out CV 2, and thus only the one-dimensional FES as a function of *z* will be discussed in the following.

**Fig. 7:**
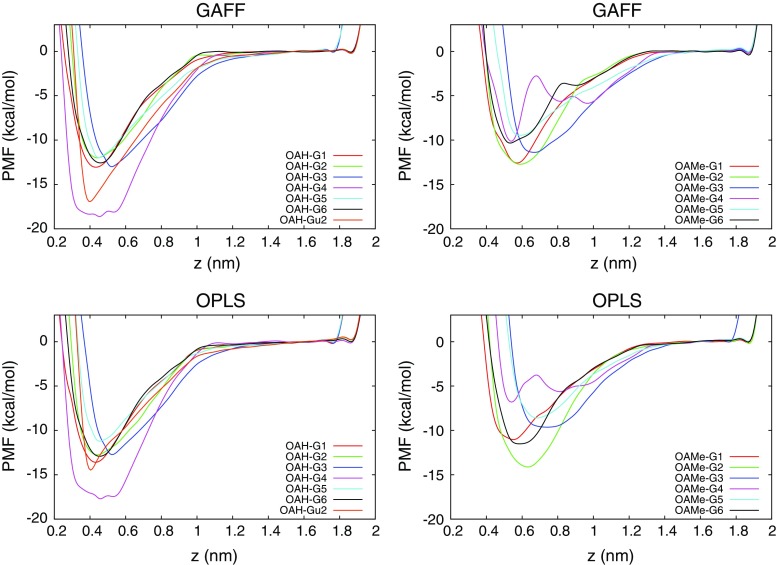
The potential of mean force for all systems and both force fields (GAFF in the *upper two panels* and OPLS in the *lower two panels*), averaged over the last 20 ns of the simulation. The arbitrary zero level has been aligned with the unbound state (so that $w(z^*)=0$) to enable an easy comparison of the PMF curves. *Error bars* for a subset of these curves are shown in Fig. S6 in the supporting information

Figure 7 shows the one-dimensional free energy surface, i.e. the potential of mean force, for each system. Error bars for four representative systems are shown in Fig. S6 in the supporting information. As already mentioned, all systems except OAMe–G4 show a single free-energy minimum and no barrier between the bound and unbound state. In general, the OAH systems show a narrower minimum and a steeper slope than the OAMe systems, for which the effective interactions (including solvent effects) seem to persist to a longer range so that the FES does not level out until $z\approx 1.3$ nm. The OAH–G4 has a significantly wider minimum than the rest of the OAH complexes, because this guest can, when bound, adopt several orientations with different values of *z* caused by the heavy bromine atom pointing in slightly different directions. It is interesting to note that G4 stands out not only computationally but also experimentally, being the worst binder to OAMe and the best binder to OAH.

The striking similarity between the results obtained using GAFF and OPLS indicates that the two force fields handle the delicate balance between the hydration of the complex and the separated monomers in a similar way (the hydrophobic effect is clearly the main driving force for binding in these systems) and validates the use of an identical computational procedure to obtain the results. The small quantitative differences between the force fields will be analyzed below.

### Comparison with experiment

**Fig. 8:**
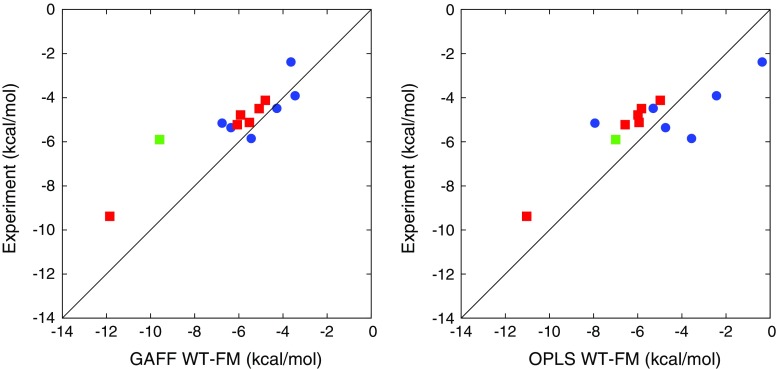
Correlation between the experimental binding free energy and the (refined) WT–FM results obtained with GAFF (*left*) or OPLS (*right*). *Blue circles* represent OAMe complexes, *red squares* represent OAH complexes, and the *green square* represents the OAH–Gu2 complex

The binding free energies calculated using WT–FM are presented in Table 2 and compared to the experimental values, defined as the average of the ITC and NMR results [24]. The experimental values were unknown at the time of submission of the *submitted results*, and ignored during the additional time needed to obtain the *refined results*, which we will mainly focus on. The correlation between the calculated and experimental values are shown in Fig. 8.

**Table 2:** Calculated $\varDelta G_ {\text {bind}}$ (kcal/mol) from WT–FM calculations using the GAFF and OPLS force fields, respectively

System	GAFF (submitted)	GAFF (refined)	OPLS (refined)	Exp
OAMe–G1	−2.56	−6.36 ± 0.39	−4.74 ± 0.52	−5.36
OAMe–G2	−2.79	−6.76 ± 0.41	−7.94 ± 0.56	−5.16
OAMe–G3	−0.84	−5.44 ± 0.56	−3.56 ± 0.45	−5.85
OAMe–G4	−0.09	−3.64 ± 1.63	−0.36 ± 0.58	−2.38
OAMe–G5	0.69	−3.45 ± 0.34	−2.43 ± 1.41	−3.91
OAMe–G6	0.05	−4.28 ± 0.34	−5.30 ± 0.70	−4.49
OAH–G1	−3.08	−6.07 ± 0.35	−6.57 ± 0.35	−5.23
OAH–G2	−2.91	−5.08 ± 0.72	−5.83 ± 0.57	−4.50
OAH–G3	−2.26	−5.92 ± 0.58	−5.99 ± 0.53	−4.79
OAH–G4	−7.94	−11.85 ± 0.61	−11.03 ± 0.61	−9.38
OAH–G5	−1.32	−4.80 ± 0.46	−4.98 ± 0.35	−4.12
OAH–G6	−3.33	−5.52 ± 0.64	−5.94 ± 0.38	−5.13
OAH–Gu2	−5.90	−9.59 ± 0.66	−7.00 ± 0.87	−5.90

Two sets of results are presented for GAFF, the difference being that the *submitted* results were obtained in shorter simulations and using an empirical correction $\varDelta G_ {\text {emp}}$ instead of a rigorous account of the water-restraint contribution $\varDelta G_ {\text {restr}}$. The estimate of the statistical error is the maximum of two error estimates: the standard deviation of $\varDelta G_ {\text {meta}}$ estimates during the last 20 ns and the absolute difference between the averages for the two last blocks of 20 ns. For the *submitted* results, the simulation time was too short to make a proper error estimate

Several error metrics are included in the SAMPL5 evaluation [24], some of which depend only on the relative free energies [the correlation coefficient ($R^2$) and the mean absolute deviation after subtraction of the average signed error (MADTr)) and others on the absolute free energies (the average signed error (AvErr) and the mean absolute deviation (MAD)]. Furthermore, the 12 systems can be evaluated in a single group (combined) or separately for the two hosts. The performance of our predictions with regards to these error metrics is shown in Table 3. For each metric, the best result among all the SAMPL5 submissions is also shown. For a more complete analysis of the submissions, we refer to Ref. [24]. To avoid being mislead by fortuitous agreement with experiment, we also evaluated the statistical distributions of the various metrics when the free-energy estimates were allowed to vary according to normal distributions with parameters from Table 2; these results are shown in Table S2 in the supporting information and it can be noted that despite some individual error estimates being rather large, the set metrics are quite stable with a typical uncertainty of $\sim$0.2 kcal/mol.

**Table 3:** Error metrics [mean absolute deviation (MAD) in kcal/mol, mean absolute deviation after translation (MADTr) in kcal/mol, average signed error (AvErr) in kcal/mol, and squared correlation coefficient ($R^2$); each calculated over OAMe and OAH separately as well as combined] for the WT–FM and MM/PBSA results using the GAFF and OPLS force fields, respectively

Metric	Group	WT–FM			MM/PBSA		Best
		GAFF (s)	GAFF	OPLS	GAFF	OPLS	
MAD	OAMe	3.60	0.82	1.67	3.88	2.29	1.44
MAD	OAH	2.05	1.02	1.20	2.39	1.48	1.13
MAD	Combined	2.82	0.92	1.44	3.14	1.89	1.51
MADTr	OAMe	1.11	0.82	1.51	3.00	2.46	0.58
MADTr	OAH	0.44	0.52	0.24	1.75	1.22	0.44
MADTr	Combined	0.94	0.64	1.31	2.38	1.77	0.58
AvErr	OAMe	3.60	−0.47	0.47	1.90	−1.23	−0.51
AvErr	OAH	2.05	−1.02	−1.20	1.93	−0.78	0.32
AvErr	Combined	2.82	−0.74	−0.37	1.91	−1.01	−0.05
$R^2$	OAMe	0.34	0.58	0.48	0.44	0.63	0.95
$R^2$	OAH	0.97	0.99	0.99	0.37	0.62	0.98
$R^2$	Combined	0.73	0.88	0.69	0.01	0.03	0.96

Two sets of results are presented for WT–FM GAFF, the difference being that the *submitted* results (s) were obtained in shorter simulations and using an empirical correction $\varDelta G_ {\text {emp}}$ instead of a rigorous account of the water-restraint contribution $\varDelta G_ {\text {restr}}$. The last column shows the best value among all SAMPL5 submissions

The empirical correction used in the SAMPL5-submitted results turned out to give poor agreement with experiment in terms of absolute binding free energies; the average signed error was 2.0–3.6 kcal/mol. This might reflect differences in the experimental procedures for the reference compound Gu2 compared to the current compounds, or some instability of the computational procedure. In contrast, the rigorous evaluation of the absolute free energies used in the refined results gave much better agreement in absolute terms, with average signed errors of 0.5–1.0 kcal/mol for GAFF and slightly larger values for OPLS, although the agreement might of course be somewhat fortuitous. Naturally, the mean absolute deviation (MAD) depends to a large extent on the successful absolute targeting; thus it is not surprising that the MAD for the refined GAFF results (0.8–1.0 kcal/mol) were better than all the SAMPL5 submissions.

It is more interesting to analyze the performance in terms of relative error metrics, because with 6 ligands (or even 12 ligands in the combined evaluation) the relative error is less prone to fortuitous error cancellation. In particular, the mean absolute deviation after translation (MADTr), i.e. after subtraction of the average signed error, has been used as an error metric for this purpose [39], although a slightly different metric was used in earlier SAMPL analyses [23]. As examplified above, the normal MAD penalizes submissions that focus on accurate relative binding free energies but have a large constant offset, whereas MADTr ignores this offset while at the same time being a more sensitive error metric than the $R^2$ correlation coefficient. Both MADTr and $R^2$ evaluate submissions of absolute and relative free energies on an even scale, and unlike the direct assessment of relative free energies, they do not depend on the choice of a reference compound.

For OAH, which is the easiest host from a sampling perspective, the submitted GAFF result has the lowest MADTr (0.44 kcal/mol) of all the SAMPL5 submissions, whereas the refined GAFF result is only slightly worse (0.52 kcal/mol, or $0.66 \pm 0.17$ kcal/mol when allowing for random errors). It is interesting to note that the second-best submission in terms of MADTr (0.64 kcal/mol) uses exactly the same force field and system setup but another free-energy method based on alchemical perturbation [40]. Moreover, the similar performance is not fortuitous; the mutual MADTr between the two submissions is only 0.22 kcal/mol, which for example is significantly less than that of changing the water model from TIP3P to OPC within the same free-energy formalism (0.57 kcal/mol) [40]. This indicates that the results of these studies are statistically converged to a level which allows force field modifications to be quantitatively assessed.

For OAMe, the results are significantly worse, with the MADTr decreasing only slightly from 1.1 to 0.8 kcal/mol between the submitted and refined results (or $0.9 \pm 0.2$ kcal/mol when allowing for random errors). The sampling problems for OAMe, especially in connection with the bulky G4 molecule, were apparent already at the time of submission and were the main motivation for continuing the WT–FM simulations to obtain refined results. However, because most of the OAMe–guest complexes are well converged, it appears that there is some sampling issue in these systems that is not readily detectable; we can rule out inaccuracy of the force field based on the good performance of the alchemical perturbation method across both hosts [40]. Nevertheless, it can be noted that the MADTr for the whole set of 12 complexes is 0.64 kcal/mol (or $0.83 \pm 0.14$ kcal/mol when allowing for random errors), which is still a good result and close to the best submission. An extension of the investigation to a larger set of guest molecules would probably be needed to further analyze the differences between the hosts.

Similarly to GAFF, the OPLS results show an excellent agreement with experiment for the OAH systems (MADTr 0.24 kcal/mol, or $0.42 \pm 0.13$ kcal/mol when allowing for random errors), but worse results than GAFF for the OAMe systems (1.5 kcal/mol) and the combined data set (1.3 kcal/mol). Again, it is impossible to point out any particular outlier in Fig. 8; we simply conclude that OAMe is a more difficult data set. To support this conclusion, one can note that only 4 of the SAMPL5 submissions for OAMe give a smaller MADTr than the “dummy prediction” that assumes equal binding free energies (0.93 kcal/mol). For OAH, the corresponding “dummy” MADTr is 1.29 kcal/mol, and as many as 14 submissions perform better than the dummy prediction; thus for e.g. force field development it makes sense to put more weight on the performance for this data set, at least until all sampling issues have been resolved.

**Fig. 9:**
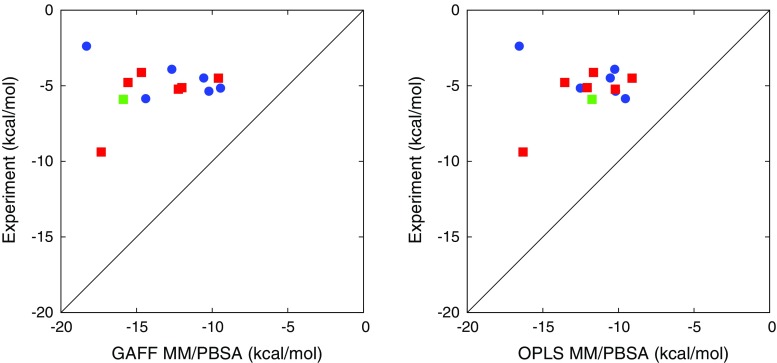
Correlation between the experimental binding free energy and the MM/PBSA estimates obtained with GAFF (*left*) or OPLS (*right*). *Blue circles* represent OAMe complexes, *red squares* represent OAH complexes, and the *green square* represents the OAH–Gu2 complex

Results of the MM/PBSA calulations are detailed in Table S3 in the supporting information and compared with experiment in Fig. 9; error metrics are presented in Table 3. As can be seen, there is no significant correlation between MM/PBSA and experiment. Interestingly, the statistical errors are always smaller than 0.2 kcal/mol, so the poor results cannot be attributed to insufficient length of the MD simulations. Instead, we must conclude that the approximations done in the employed version of MM/PBSA are not sufficiently accurate to describe the subtle differences in binding free energies between the various ligands. This is somewhat expected for this type of systems, which are not dominated by strong energetic interactions. In particular, the assumption of equal conformational entropy loss for all ligands is probably quite crude, and the Poisson–Boltzmann (PB) implicit-solvent model might have difficulties with capturing the discrete-water effect of a narrow binding pocket. To test the sensitivity of the results to the parameters of the PB model, we calculated the binding free energies using three different choices of atomic radii (the “Bondi”, “Mbondi”, and “Mbondi2” settings, respectively) and, for Bondi radii, three choices of the solute dielectric constant ($\epsilon _ {\text {solute}} =1$, 2, and 4, respectively). The change of radii had a significant effect only on the results involving G4, for which the change of the bromine atomic radius shifted the binding free energies with 1.6–2.0 kcal/mol in the positive direction. On the other hand, the change of $\epsilon _ {\text {solute}}$ gave a mean absolute deviation of 2.8–3.0 kcal/mol (when going from $\epsilon _ {\text {solute}} =1$ to 2) or 4.3–4.5 kcal/mol (when going from $\epsilon _ {\text {solute}} =1$ to 4), but this is mainly due to a constant offset; the MADTr for the same changes is 0.7 and 1.1 kcal/mol, respectively. Thus, we can conclude that the relative binding free energy predictions are stable with respect to the PB parameters, and therefore that a particular choice of PB parameters is not responsible for the poor agreement with experiment.

### Force field comparison

**Fig. 10:**
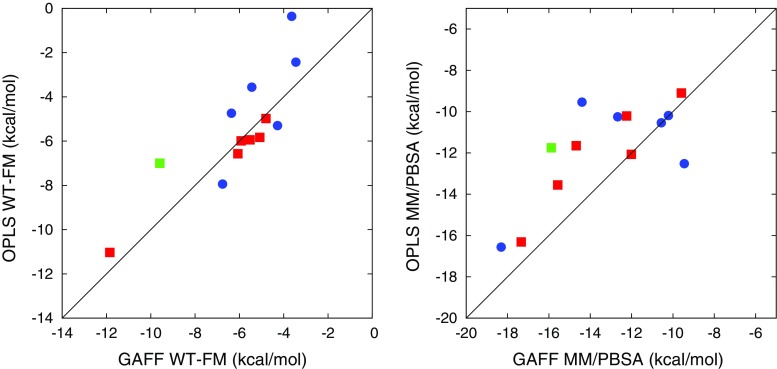
Correlation between GAFF and OPLS results obtained by WT–FM (*left*) and MM/PBSA (*right*). *Blue circles* represent OAMe complexes, *red squares* represent OAH complexes, and the *green square* represents the OAH–Gu2 complex

The left part of Fig. 10 compares the WT–FM binding free energies obtained with GAFF and OPLS, respectively. As can be seen, the agreement is almost perfect for OAH. In contrast, the OAMe results are much more scattered. To some extent, this apparent force field discrepancy probably reflects the sampling problems mentioned above. Indeed, the most prominent outlier is the problematic OAMe–G4 complex, which is predicted to bind much more strongly with GAFF than with OPLS. Proving the presence of a significant force field discrepancy would require running several independent WT–FM simulations with each force field and performing a proper statistical analysis of the variation.

In the right part of Fig. 10, the corresponding information is shown for the MM/PBSA results. Again, the agreement is better for OAH than for OAMe, but for both hosts it is significantly worse for MM/PBSA than for WT–FM. Apparently, there is an additional contribution to the force field discrepancy coming from the use of the approximate MM/PBSA method (note that it is not random noise because the MM/PBSA results are well converged). Within the MM/PBSA framework, we analyzed which term contributed most to the discrepancy and found, for both hosts, rather similar contributions from the Van der Waals energy on one hand (on average 1.8 kcal/mol) and from the sum of electrostatics and PB energy on the other hand (1.9 kcal/mol), but in the case of OAH, these contributions partly cancel out and thereby give a smaller overall discrepancy. However, owing to the poor overall agreement with experimental data, it is questionable how trustworthy such detailed analysis of the MM/PBSA energy terms actually is.

## Conclusions

We have used well-tempered funnel metadynamics to compute binding free energies for a set of six guest molecules binding to the octa-acid host (OAH) and the same set binding to the similar OAMe host. The aim of this investigation was to refine the computational procedure for applying funnel metadynamics and possibly reduce the computational cost of the method, but also to evaluate both the free-energy methodology and the chosen force field in the SAMPL5 blind challenge, as well as testing the much simpler MM/PBSA approach for these systems.

With regards to the computational procedure, we found that two collective variables (host–guest distance and guest orientation) are needed to enhance the sampling. However, the re-entry of the guest into the host is hindered by the presence of trapped water molecules inside the host. The water molecules would have to leave for the guest molecule to be able to bind, thus creating a clash in the narrow binding channel. The kinetic barrier connected to this desolvation created a hysteresis-like effect that prevented the smooth convergence of the free-energy surface. The occurrence of such barrier depends on the geometry of the binding site; for example, it was naturally not observed for a ring-shaped host that is open in both ends [13], but has been observed in protein–ligand systems, where specially developed collective variables that explicitly take the solvent molecules into account have been proposed [9].

We have herein proposed and tested a much simpler way to avoid the problem, namely to divide the binding process into a desolvation step and an actual binding step. The free energy of the desolvation step can be computed using a rigorous FEP approach, in general requiring several intermediates but in simple cases (like the one presented here) requiring only an unbiased MD simulation of the host. The free energy of the binding step can be calculated by funnel metadynamics, but using a water restraint that prevents water molecules from entering the deeper part of the binding site where they might get trapped. For most systems tested in this study, a smooth convergence of the binding free energy prediction was then obtained within 30–40 ns of simulation. It remains to investigate whether the method is applicable to deeper binding cavities, or whether a more elegant but costly approach with a specific collective variable for the solvation is more appropriate for such systems.

With regards to the accuracy test, we found that the converged free energies agreed well with experiment, especially for the OAH host, for which the results were among the best submissions to the SAMPL5 challenge when evaluated in terms of relative binding free energies. In contrast, MM/PBSA gave poor results with no predictivity. The good results obtained with funnel metadynamics validates both the free-energy method and the employed force field, which was a standard GAFF/TIP3P combination. It might seem strange that a standard force field provides reliable results for such unusual molecules, but similar good results were in fact obtained previously for this host using the same force field [41]. Of course, the performance of force fields for this type of molecules is highly system-dependent and more careful studies are needed to investigate the performance of various force fields. Our study has shown that funnel metadynamics can provide sufficiently high statistical precision to adequately address such problems in the future.

## Electronic supplementary material

Below is the link to the electronic supplementary material.

Supplementary material 1 (pdf 927 KB)
