# Supplementary material for: Resolving the problem of trapped water in binding cavities: prediction of host–guest binding free energies in the SAMPL5 challenge by funnel metadynamics
Source: J Comput Aided Mol Des. 2016 Aug 29;31(1):119–32. doi: 10.1007/s10822-016-9948-6 (PMC5239820; doi:10.1007/s10822-016-9948-6)
Supplement: Supplementary file 1 — Supplementary material 1 (pdf 927 KB) [file 10822_2016_9948_MOESM1_ESM.pdf]

Supporting information

S. Bhakat and P. Söderhjelm

Resolving the problem of trapped water in binding cavities: Prediction of host-guest binding free energies in the SAMPL5 challenge by funnel metadynamics

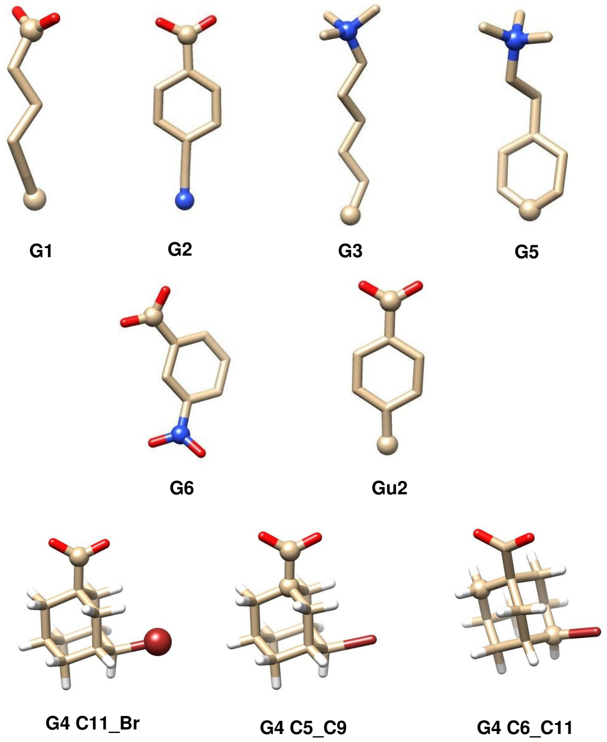

Fig. S1 3D representation of the guest molecules. For each guest molecule, the two atoms defining the guest molecular axis are shown as balls. Due to its asymmetrical nature of G4, three different atom pairs were tested in order to define the molecular axis for this ligand.

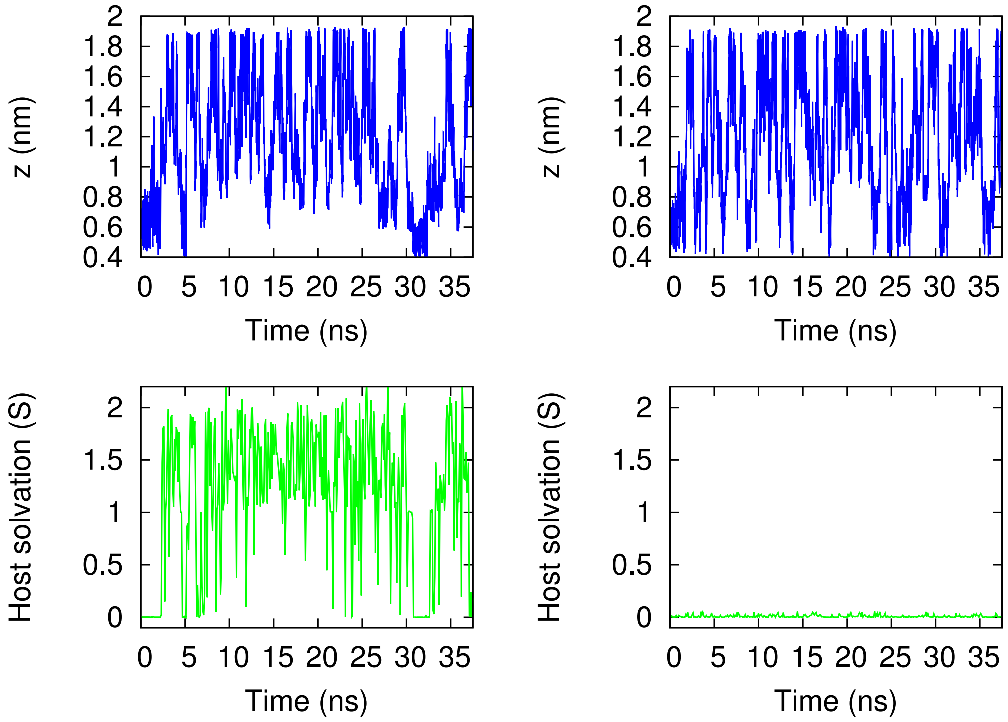

Fig. S2 Comparison of the beginnings of the metadynamics trajectories of OAMe–G2 without water restraint (left) and with water restraint (right), but otherwise with the same settings. Both the position of the guest molecule (z; blue) and the host solvation (S; green) is shown.

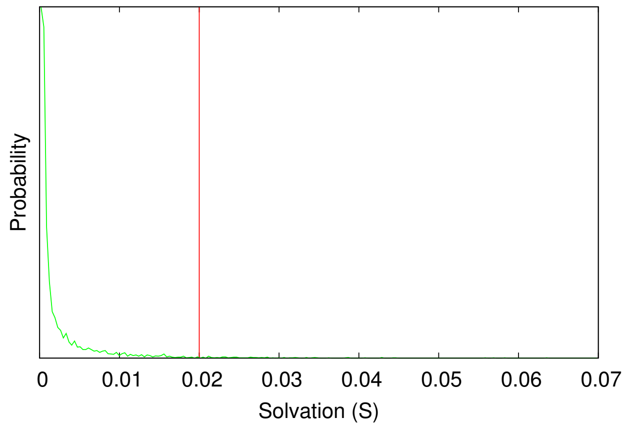

Fig. S3 Probability distribution of the solvation number (S) in unrestrained simulations of the OAMe–G2 complex. The vertical line marks the value S_0, above which the water restraint sets in (see Eq. 3).

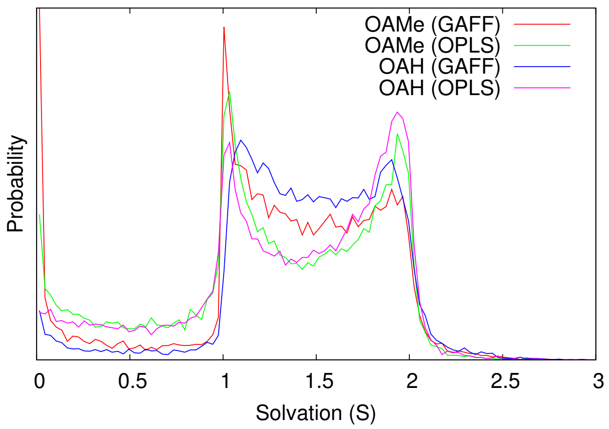

Fig. S4 Probability distribution of the solvation number (S) in unrestrained simulations of the solvated host molecule. The value of S roughly corresponds to the number of water molecules deep inside the host. Data from two different hosts and two different force fields are shown.

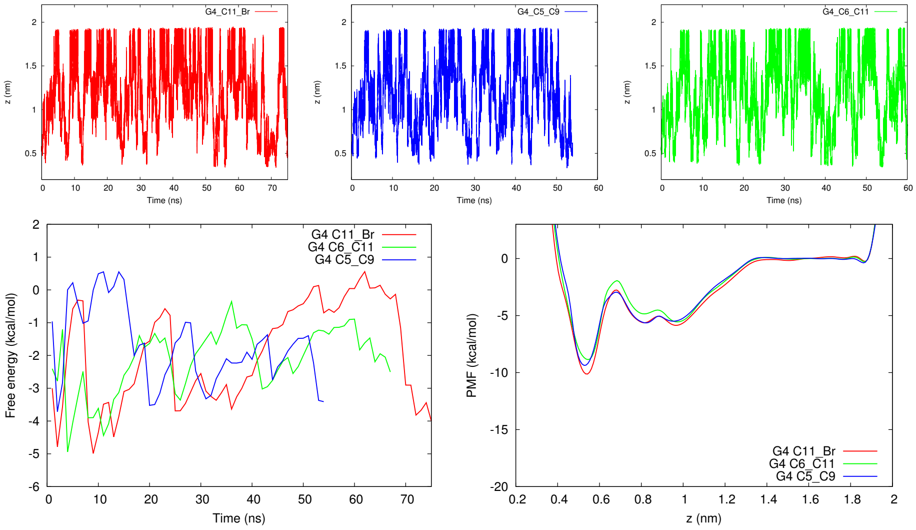

Fig. S5 Investigation of the effect of the choice of guest axis on the convergence behavior of the OAMe–G4 complex using the GAFF force field. The upper row of figures show the time evolution of CV 1 (projection of the ligand on the z axis) for three different choices of axes used for CV 2 (cf. Fig. S1), with the first being the one used throughout this article. The lower left figure shows the convergence of free energies with the same three choices. The lower right figure shows the one-dimensional free energy obtained from each simulation, averaged over the last 20 ns. Both G4 C6_C11 and G4 C5_C9 show more recrossings within 50 ns compared to G4 C11_Br. Whereas G4 C6_C11 shows slightly more recrossings compared to G4 C5_C9, more simulation time is needed before any definitive conclusions are drawn. It is reassuring that the PMF is practically identical for the different choices of guest axis; only the convergence behavior is affected.

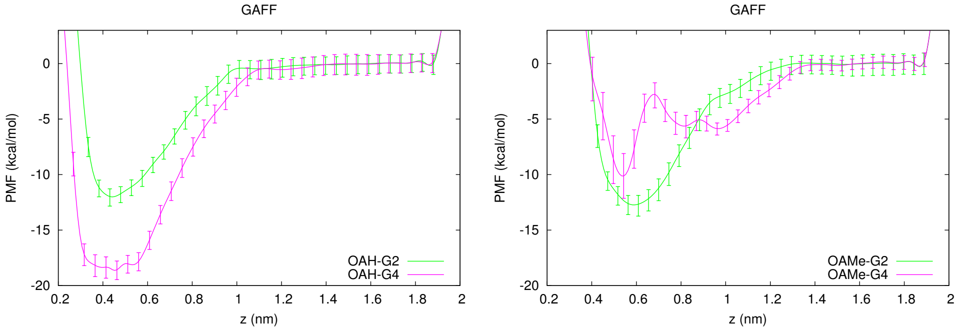

Fig. S6 The potential of mean force for four representative systems evaluated with GAFF, averaged over the last 20 ns of the simulation and including error bars defined as the standard deviation over the same interval.

Table S1 Time evaluation of the estimated ΔG_{meta} in kcal/mol. The values are block averages over a set of estimates in a 10 or 20 ns period and the precision is approximated by the fluctuation (standard deviation) of this estimate in each period (note that the standard error of the mean in not applicable here because the set of estimates are not independent samples of an underlying distribution). The *last 20 ns* period was used in the error analysis in this article. For GAFF, the estimates of the first period (30–40 ns) coincide with the SAMPL5-submitted results, except for an empirical constant ΔG_{emp}. The Diff column gives the difference between the averages over the *last 20 ns* and the 30–50 ns period.

System	30–40 ns	40–50 ns	50–60 ns	60–70 ns	Last 20 ns	Diff
GAFF:						
OAMe–G1	-8.42 ± 0.30	-8.61 ± 0.28	-8.63 ± 0.41	-8.21 ± 0.37	-8.12 ± 0.33	0.39
OAMe–G2	-8.65 ± 0.15	-7.58 ± 0.55	-8.68 ± 0.31	-8.31 ± 0.38	-8.52 ± 0.37	-0.41
OAMe–G3	-6.70 ± 0.32	-6.59 ± 0.39	-7.00 ± 0.58	-6.90 ± 0.08	-7.20 ± 0.22	-0.56
OAMe–G4	-5.95 ± 0.29	-3.90 ± 0.69	-2.92 ± 0.31	-2.96 ± 0.93	-5.40 ± 1.63	-0.48
OAMe–G5	-5.17 ± 0.55	-5.31 ± 0.50	-5.63 ± 0.30	-5.35 ± 0.26	-5.21 ± 0.34	0.03
OAMe–G6	-5.81 ± 0.44	-5.81 ± 2.71	-5.19 ± 0.28	-5.63 ± 0.50	-6.04 ± 0.34	-0.23
OAH–G1	-8.94 ± 0.40	-8.86 ± 0.44	-8.79 ± 0.47	-8.76 ± 0.28	-8.74 ± 0.35	0.17
OAH–G2	-8.77 ± 0.33	-8.17 ± 0.41	-7.84 ± 0.48	-7.57 ± 0.20	-7.75 ± 0.38	0.72
OAH–G3	-8.12 ± 0.41	-7.90 ± 0.31	-7.94 ± 0.58	-8.47 ± 0.16	-8.59 ± 0.33	-0.58
OAH–G4	-13.80 ± 0.57	-14.01 ± 0.29	-14.39 ± 0.71	-14.57 ± 0.65	-14.52 ± 0.61	-0.61
OAH–G5	-7.18 ± 0.32	-6.99 ± 0.37	-7.11 ± 0.24	-7.41 ± 0.45	-7.47 ± 0.46	-0.38
OAH–G6	-9.19 ± 0.52	-8.46 ± 0.46	-7.84 ± 0.26	-8.53 ± 0.35	-8.19 ± 0.48	0.64
OAH–Gu2	-11.76 ± 0.49	-12.59 ± 0.47	-11.72 ± 0.51	-12.64 ± 0.37	-12.25 ± 0.66	-0.08
OPLS:						
OAMe–G1	-7.10 ± 0.60	-7.46 ± 0.46	-7.02 ± 0.42	-6.79 ± 0.29	-6.76 ± 0.23	0.52
OAMe–G2	-9.06 ± 0.46	-9.73 ± 0.27	-9.83 ± 0.71	-10.04 ± 0.35	-9.96 ± 0.49	-0.56
OAMe–G3	-5.47 ± 0.37	-4.90 ± 0.47	-6.02 ± 0.25	-5.26 ± 0.23	-5.58 ± 0.45	-0.40
OAMe–G4	-1.70 ± 0.66	-1.92 ± 0.41	-2.43 ± 0.28	-1.96 ± 0.37	-2.38 ± 0.38	-0.58
OAMe–G5	-3.38 ± 0.46	-2.70 ± 0.42	-3.11 ± 0.42	-4.50 ± 0.33	-4.45 ± 0.73	-1.41
OAMe–G6	-5.93 ± 0.72	-7.32 ± 0.69	-7.60 ± 0.25	-7.23 ± 0.44	-7.32 ± 0.36	-0.70
OAH–G1	-8.83 ± 0.33	-9.06 ± 0.30	-8.43 ± 0.26	-8.82 ± 0.50	-9.20 ± 0.35	-0.25
OAH–G2	-7.81 ± 0.37	-7.97 ± 0.34	-7.77 ± 0.42	-8.76 ± 0.21	-8.46 ± 0.39	-0.57
OAH–G3	-8.22 ± 0.40	-8.34 ± 0.50	-8.79 ± 0.41	-8.80 ± 0.32	-8.62 ± 0.53	-0.34
OAH–G4	-14.48 ± 0.50	-13.26 ± 0.42	-13.74 ± 0.40	-13.35 ± 0.61	-13.66 ± 0.61	0.21
OAH–G5	-7.15 ± 0.86	-7.42 ± 0.24	-7.47 ± 0.35	-7.87 ± 0.25	-7.61 ± 0.35	-0.33
OAH–G6	-8.34 ± 0.55	-8.05 ± 0.32	-8.27 ± 0.25	-8.69 ± 0.18	-8.57 ± 0.34	-0.38
OAH–Gu2	-9.04 ± 0.93	-9.31 ± 0.37	-8.84 ± 0.65	-10.30 ± 0.45	-9.62 ± 0.87	-0.45

Table S2 Statistical evaluation of error metrics (mean absolute deviation (MAD) in kcal/mol, mean absolute deviation after translation (MADTr) in kcal/mol, average signed error (AvErr) in kcal/mol, and squared correlation coefficient (R^2); each calculated over OAMe and OAH separately as well as combined) for the refined WT-FM calculations using the GAFF and OPLS force fields, respectively. Reported values are the mean and standard deviation of the distribution of each metric when the calculated free energies are allowed to vary according to normal distributions with parameters from Table 2. The results were obtained through a bootstrapping procedure using 1000 samples.

Metric	Group	GAFF	OPLS
MAD	OAMe	0.94 ± 0.23	1.73 ± 0.27
MAD	OAH	1.08 ± 0.20	1.21 ± 0.20
MAD	combined	1.01 ± 0.15	1.46 ± 0.17
MADTr	OAMe	0.92 ± 0.23	1.61 ± 0.24
MADTr	OAH	0.66 ± 0.17	0.42 ± 0.13
MADTr	combined	0.83 ± 0.14	1.34 ± 0.18
AvErr	OAMe	-0.47 ± 0.31	0.47 ± 0.31
AvErr	OAH	-1.01 ± 0.23	-1.21 ± 0.20
AvErr	combined	-0.74 ± 0.19	-0.37 ± 0.18
R^2	OAMe	0.50 ± 0.27	0.45 ± 0.09
R^2	OAH	0.95 ± 0.03	0.95 ± 0.03
R^2	combined	0.81 ± 0.11	0.66 ± 0.06

Table S3 MM/PBSA results in kcal/mol. The total binding free energy is divided into the MM interaction energy (subdivided into electrostatic and van der Waals contribution), the Poisson–Boltzmann solvation free energy, and the non-polar solvation free energy. Standard error of the mean for all terms are also reported.

System	Vdw	Ele	PB	non-polar	Total
GAFF:					
OAMe–G1	-19.85 ± 0.03	221.55 ± 0.07	-209.90 ± 0.07	-2.02 ± 0.00	-10.22 ± 0.04
OAMe–G2	-19.97 ± 0.04	222.03 ± 0.11	-209.53 ± 0.12	-1.99 ± 0.00	-9.45 ± 0.08
OAMe–G3	-20.03 ± 0.04	-214.13 ± 0.10	222.05 ± 0.11	-2.29 ± 0.00	-14.40 ± 0.07
OAMe–G4	-25.03 ± 0.03	218.53 ± 0.06	-209.48 ± 0.05	-2.33 ± 0.00	-18.31 ± 0.03
OAMe–G5	-21.61 ± 0.04	-222.78 ± 0.10	234.02 ± 0.11	-2.30 ± 0.00	-12.67 ± 0.12
OAMe–G6	-21.43 ± 0.04	225.39 ± 0.08	-212.46 ± 0.07	-2.06 ± 0.00	-10.56 ± 0.04
OAH–G1	-20.36 ± 0.02	231.46 ± 0.07	-221.60 ± 0.06	-1.74 ± 0.00	-12.24 ± 0.03
OAH–G2	-21.17 ± 0.04	232.12 ± 0.11	-218.85 ± 0.08	-1.69 ± 0.00	-9.59 ± 0.02
OAH–G3	-21.33 ± 0.03	-228.46 ± 0.15	236.27 ± 0.17	-2.04 ± 0.00	-15.57 ± 0.11
OAH–G4	-26.48 ± 0.04	227.66 ± 0.11	-216.43 ± 0.07	-2.09 ± 0.00	-17.34 ± 0.05
OAH–G5	-22.40 ± 0.03	-237.36 ± 0.14	247.13 ± 0.15	-2.05 ± 0.00	-14.68 ± 0.08
OAH–G6	-22.09 ± 0.03	231.20 ± 0.07	-219.30 ± 0.06	-1.83 ± 0.00	-12.01 ± 0.03
OAH–Gu2	-20.94 ± 0.02	231.51 ± 0.08	-224.71 ± 0.07	-1.74 ± 0.00	-15.88 ± 0.03
OPLS:					
OAMe–G1	-20.40 ± 0.03	207.76 ± 0.09	-195.54 ± 0.07	-2.02 ± 0.00	-10.19 ± 0.14
OAMe–G2	-19.54 ± 0.04	200.37 ± 0.10	-191.37 ± 0.08	-1.98 ± 0.00	-12.52 ± 0.06
OAMe–G3	-16.98 ± 0.04	-194.00 ± 0.09	203.61 ± 0.12	-2.18 ± 0.00	-9.54 ± 0.10
OAMe–G4	-22.89 ± 0.12	200.39 ± 0.15	-191.65 ± 0.14	-2.41 ± 0.01	-16.56 ± 0.13
OAMe–G5	-18.54 ± 0.04	-195.12 ± 0.10	205.58 ± 0.13	-2.17 ± 0.00	-10.25 ± 0.04
OAMe–G6	-19.97 ± 0.06	203.32 ± 0.11	-191.84 ± 0.09	-2.05 ± 0.00	-10.54 ± 0.05
OAH–G1	-20.67 ± 0.03	234.09 ± 0.39	-221.77 ± 0.30	-1.87 ± 0.00	-10.21 ± 0.08
OAH–G2	-19.59 ± 0.04	224.52 ± 0.35	-212.20 ± 0.28	-1.83 ± 0.00	-9.10 ± 0.08
OAH–G3	-22.71 ± 0.04	-263.30 ± 0.46	274.92 ± 0.50	-2.47 ± 0.00	-13.55 ± 0.05
OAH–G4	-22.69 ± 0.03	209.76 ± 0.10	-201.21 ± 0.07	-2.17 ± 0.00	-16.31 ± 0.04
OAH–G5	-19.14 ± 0.04	-209.80 ± 0.12	219.32 ± 0.14	-2.04 ± 0.00	-11.65 ± 0.06
OAH–G6	-20.47 ± 0.03	213.55 ± 0.08	-203.25 ± 0.07	-1.90 ± 0.00	-12.07 ± 0.05
OAH–Gu2	-20.80 ± 0.03	217.11 ± 0.09	-206.18 ± 0.09	-1.87 ± 0.00	-11.75 ± 0.05

Table S4 Plumed 1.3 input file for the WT-FM simulation of OAH–G1.

```
# Plumed 1.3 input file for OAH--G1
# Intended for GROMACS, thus units are nanometers and kJ/mol
# Compatible with the SAMPL5-provided GROMACS simulation files
# after adding a virtual atom with index 185 on the midpoint between atoms 29 and 2.
#####################################################################################
#The main CV: z
DISTANCE LIST <ax0> <ax1> PROJ_ON_AXIS <lig> SIGMA 0.04 NOPBC
#The distance from the z axis used to define the funnel potential
DISTANCE LIST <ax0> <ax1> POINT_FROM_AXIS <lig> NOPBC

#The orientation CV is computed as W=(Z1-Z2)/R12
#where Z1 and Z2 are the projections of the two chosen ligand atoms on the z axis,
#and R12 is the maximum distance between them obtained in a typical MD simulation.
#Thus we first define the two CVs Z1 and Z2, and then use the POLY construction to combine them
DISTANCE LIST <ax0> <ax1> PROJ_ON_AXIS 186 NOPBC
DISTANCE LIST <ax0> <ax1> PROJ_ON_AXIS 191 NOPBC
POLY TERMS 2 SIGMA 0.043
CV 3 COEFF 1.79706
CV 4 COEFF -1.79706

#Coordination number keyword
COORD LIST 185 <solvent> NN 16 MM 32 D_0 0.0 R_0 0.35

#To avoid PBC problems caused by domain decomposition in Gromacs, we use ALIGN_ATOMS to ensure that the
#relevant host and guest atoms obey the minimum-image convention.
ALIGN_ATOMS LIST <host_guest>
#Define walls on the main CV and on the coordination number.
#and read in funnel potential created by a separate script
LWALL CV 1 LIMIT -1.85 KAPPA 500000.0 EXP 4
UWALL CV 1 LIMIT  1.85 KAPPA 500000.0 EXP 4
UWALL CV 6 LIMIT 0.02 KAPPA 2000000.0 EXP 4
EXTERNAL NCV 2 CV 1 2 FILENAME funnel.dat

#Define metadynamics on CV 1 and 5
HILLS RESTART HEIGHT 0.5 W_STRIDE 500
WELLTEMPERED SIMTEMP 300 BIASFACTOR 20
NOHILLS CV 2
NOHILLS CV 3
NOHILLS CV 4
NOHILLS CV 6

#Define atom groups
ax0-> 34 28 21 14 9 5 40 30 7 10 17 22 23 26 32 33 ax0<-
ax1-> 104 70 90 64 98 58 125 76 81 82 83 84 85 86 87 94 ax1<-
lig-> LOOP 186 193 1   lig<-
host-> LOOP 1 128 1   host<-
solvent->   LOOP 210 6509 3    solvent<-
host_guest->   34 28 21 14 9 5 40 30 7 10 17 22 23 26 32 33 104 70 90 64 98 58
125 76 81 82 83 84 85 86 87 94 186 187 188 189 190 191 192 193  host_guest<-
ENDMETA
```
